# Dysregulated T cell responses and inflammatory cytokine profiles in patients with acute chikungunya fever: a study from Guangdong, 2025

**DOI:** 10.3389/fcimb.2026.1774254

**Published:** 2026-04-07

**Authors:** Mingya Xiao, Zijing Liu, Si Wang, Qian Yi, E-ying Lu, Ruirong Xu, Wei Li, Yongkui Li, Wenli Li

**Affiliations:** 1 Department of Infectious Diseases, The Affiliated Guangdong Second Provincial General Hospital of Jinan University, Guangzhou, China; 2 Department of Immunology and Microbiology, Institute of Medical Microbiology, College of Life Science and Technology, Jinan University, Guangzhou, China; 3 Key Laboratory of Viral Pathogenesis & Infection Prevention and Control (Jinan University), Ministry of Education, Guangzhou, China

**Keywords:** arthralgia, chikungunya virus, fever, IL-1β, inflammatory cytokines, T cell dysregulation

## Abstract

Chikungunya fever (CHIKF), an acute arboviral disease caused by the chikungunya virus (CHIKV), is characterized by fever, debilitating arthralgia, and systemic inflammation, posing a significant public health burden in endemic regions like Guangdong Province, China. The early immunopathological mechanisms following CHIKV infection remain incompletely understood. This prospective cohort study investigated dysregulated effector T cell responses and inflammatory cytokine profiles in 34 patients with acute CHIKF, compared to 20 healthy controls, during a 2025 outbreak in Guangdong. Flow cytometry and multiplex cytokine analysis revealed significant suppression of key effector populations, including CD3^+^ T cells, CD8^+^ T cells, and natural killer T (NKT) cells, alongside a marked elevation of pro−inflammatory cytokines IL−1β, IL−6, and IL−8. The degree of T cell dysregulation is closely related to the appearance of clinical symptoms, particularly arthralgia and fever. Furthermore, pre−existing comorbidities and advanced age were associated with more pronounced immune abnormalities. Male patients exhibited a higher risk of inflammatory dysregulation, demonstrated by greater NKT cell depletion and IL−1β upregulation compared to females. Notably, pro−inflammatory cytokine levels strongly correlated with neutrophil counts and systemic inflammatory markers but not with T cell subset alterations, suggesting distinct pathological pathways. These findings delineate a dual immunopathogenic state in acute CHIKV infection, involving concurrent effector T cell suppression and IL-1β-associated inflammatory response, which provides insights into potential biomarkers and therapeutic targets for disease management.

## Introduction

Chikungunya fever (CHIKF) is a mosquito−borne arboviral disease caused by the chikungunya virus (CHIKV), a single−stranded positive−sense RNA virus belonging to the genus Alphavirus, family Togaviridae (Freppel et al., 2024). The virus is primarily transmitted by Aedes aegypti and Aedes albopictus mosquitoes and manifests as an acute febrile illness accompanied by rash, debilitating arthralgia, and myalgia, often resembling rheumatoid arthritis (Paul and Sadanand, 2018; Vairo et al., 2019). Since its first identification in Tanzania in 1952, CHIKV has caused periodic outbreaks in Africa, Asia, Europe, and the Americas, posing a substantial public health burden (Ross, 1956; Bettis et al., 2022; Costa et al., 2023; Chikungunya epidemiology update - June 2025, 2025). In China, local transmissions have been reported in several provinces including Guangdong, Zhejiang, and Yunnan, with an ongoing outbreak documented in Foshan in 2025 (Zhang et al., 2025).

The pathogenesis of CHIKV involves both direct viral cytopathy and host immune responses. Upon infection, CHIKV triggers innate immune activation through pattern recognition receptors (PRRs), leading to the production of type I interferons and pro−inflammatory cytokines (Teng et al., 2012; Haese et al., 2016). Notably, the inflammasome complex, which orchestrates the maturation and secretion of IL−1β and IL−18, has been implicated in the cytokine storm observed in severe viral infections (Ng et al., 2009; Chaaitanya et al., 2011; Chow et al., 2011). In CHIKV infection, excessive release of cytokines such as IL−1β, IL−6, and IL−8 may contribute to tissue damage and clinical symptomatology (Hoarau et al., 2010; Lohachanakul et al., 2012; Reddy et al., 2014).

Adaptive immunity, particularly T cell responses, plays a crucial role in viral clearance and immunopathology. Studies in murine models suggest that CD4^+^ T cells contribute to joint inflammation, whereas CD8^+^ T cells are essential for viral control (Wauquier et al., 2011; Teo et al., 2013; Henderson Sousa et al., 2023; Bullock et al., 2024). However, human data remain inconsistent (Hawman et al., 2013; Ruiz Silva et al., 2016; Agarwal et al., 2025a), and the interplay between T cell dysfunction and inflammasome activation in acute CHIKF is not fully understood. Moreover, most previous cytokine profiles were derived from outbreaks before 2010, and data from recent Chinese epidemics are scarce.

In this study, we profiled the immune responses of 34 patients with acute CHIKF during the 2025 outbreak in Guangdong. Using multiparametric flow cytometry and cytokine quantification, we evaluated the alterations in T cell subsets and inflammatory mediators and correlated them with clinical features. Our findings underscore the simultaneous suppression of effector T cells and upregulation of inflammasome−associated cytokines, providing a clinical perspective on the immunopathology of current CHIKV infection.

## Materials and methods

### Research approval

This study was approved by the Ethics Committee of the Second People’s Hospital of Guangdong Province (Ethical Approval No. 2025-KY-KZ-424-01). The investigation was conducted in Guangdong Province in strict compliance with the ethical standards of the Declaration of Helsinki and the International Council for Harmonisation (ICH) Good Clinical Practice guidelines. Prior to inclusion, written informed consent was secured from every participant or, when applicable, their legal guardians. To safeguard participant confidentiality, all biological samples were irreversibly de-identified and assigned unique study identifiers immediately following collection.

### Clinical sample collection

Thirty-four participants diagnosed with CHIKV during the August 2025 outbreak in Guangdong Province were recruited. Chikungunya virus infection was confirmed using nucleic acid testing. No medication was administered prior to sample collection. Samples were collected at the hospital upon the onset of infection-related symptoms, all within three days. Patients were hospitalized for treatment following sample collection. From each participant, 5 mL of venous blood was collected within three days of symptom onset. All participants were confirmed to be negative for dengue virus infection through ELISA and rapid antibody diagnostic tests. Clinical data, including fever duration, arthralgia, rash, and underlying medical conditions, were documented at enrollment. In addition, blood samples were collected from 20 age- and sex-matched healthy controls, who had not taken any medication or sought medical treatment for illness within the past month.

### Flow cytometry for immunotyping of immune cells and detecting of cytokines

For flow cytometry, whole blood was drawn into citrate-anticoagulant vacuum tubes. For immunophenotyping by flow cytometry, the whole blood was first stained with fluorochrome-conjugated antibodies. Following surface staining, erythrocytes were lysed using a dedicated red blood cell lysis buffer. The leukocyte pellet was then collected by centrifugation, washed, and prepared for acquisition. Specifically, 100 μL aliquots of whole blood were incubated with pre-mixed antibody cocktails (2–3 antibodies per panel) targeting various cell surface markers. Antibodies were conjugated to ECD (R-Phycoerythrin-Texas Red), PE (R-Phycoerythrin), or FITC (Fluorescein Isothiocyanate). A combination of 12 antibodies was used, including CD45-ECD (Beckman Coulter, Villepinte, France, A07784)/CD14-PE (Beckman Coulter, A07764);HLA-DR-ECD (Beckman Coulter, IM3636)/CD11c-PE (Beckman Coulter, IM1760)/Lin 1-FITC (BD Biosciences, San Jose, CA, 340546); HLA-DR-ECD (Beckman Coulter, IM3636)/CD80-PE (Beckman Coulter, 1976)/Lin 1-FITC (BD Biosciences, 340546);CD3-ECD (Beckman Coulter A07748)/CD4-PE (Beckman Coulter, A07751)/CD8-FITC (Beckman Coulter, A07756);HLA-DR-ECD (Beckman Coulter, IM3636)/CD4-PE (Beckman Coulter, A07751)/CD8-FITC (Beckman Coulter, A07756);CD3-ECD (Beckman Coulter, A07748)/TCR Pan α/β-PE (Beckman Coulter, 1467)/TCR Pan γδ-FITC (Beckman Coulter, 1571);CD19-ECD (Beckman Coulter, A07770)/IgD-PE (BD Biosciences, 555779)/CD27-FITC (BD Biosciences, 555440);CD19-ECD (Beckman Coulter, A07770)/CD5-PE (Beckman Coulter, A07753)/CD23-FITC (Beckman Coulter, IM0529); CD3-ECD (Beckman Coulter, A07748)/CD56-PE (Beckman Coulter, A07788)/CD69-FITC (BD Biosciences, 555530). The antibody dilution and dosage for each sample were used strictly in accordance with the manufacturer’s instructions.The gating strategy and quality control are shown in **Supplementary Figure 1**, which displays the results after compensation.

The twelve-factor (IL-2, IL-4, IL-6, IL-10, TNF-α, IFN-γ, IL-17A, IL-1β, IL-5, IL-12p70, IFN-α, IL-8) detection kit (Biorbyt Company, Guangzhou, KG0475) is purchased from Shenzhen Huize Biotechnology Co., LTD. After treatment with Beckman Coulter’s ImmunoPrep Reagent Systems at the TQ-Prep workstation (Beckman Coulter EPICS XL-MCL flow cytometer). The samples were analyzed using System II acquisition software and CXP analysis software (version 2.2).

### Statistical analysis

All data were subjected to normality and homogeneity of variance tests. For data that met the normal distribution and homogeneity of variance criteria, T-tests were employed; for the remaining data, non-parametric Mann-Whitney U tests (one-tailed) were conducted. For comparisons between the healthy control group and patients with chikungunya fever, the non-parametric Mann-Whitney U test was used. Additionally, within the patient group, comparisons were made among the following groups: (1) male patients versus female patients; (2) patients younger than 50 years versus those 50 years or older; (3) patients with underlying diseases versus those without; (4) patients with joint pain versus those without; (5) patients with fever symptoms versus those without; (6) patients with rashes versus those without. Spearman correlation analysis was used to assess the correlations among blood parameters, immune cell counts, and cytokine levels, with a correlation coefficient (r) greater than 0.4 defined as a strong correlation. Chi-square tests were used to analyze categorical data. All statistical analyses were performed using SPSS 27, and graphs were generated using GraphPad Prism 9 (scatter plots) and OriginPro 2024b (Spearman correlation heatmaps).

## Results

### CHIKF patients exhibit suppression in effector T cells and elevated pro-inflammatory cytokines

**Figure 1:**
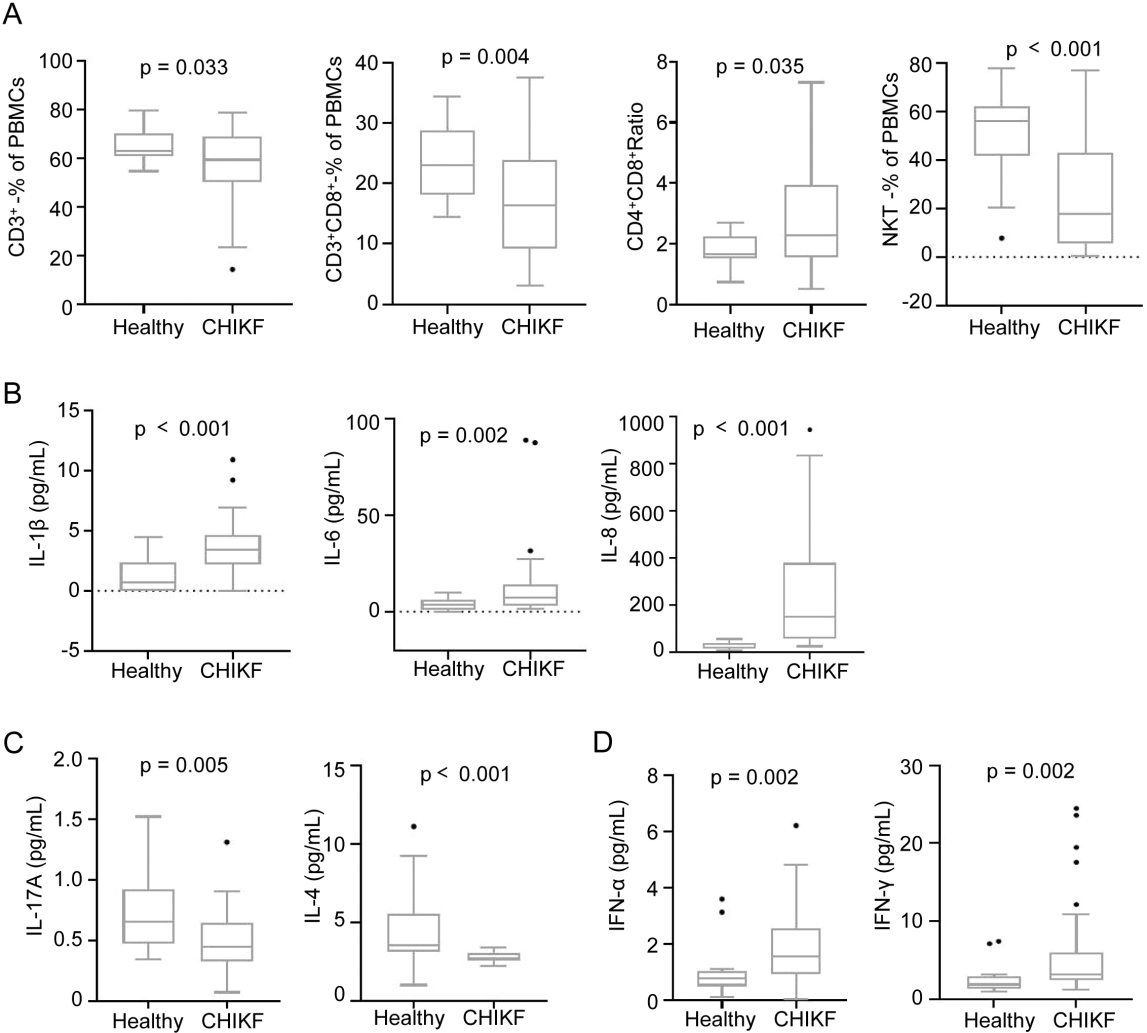
Comparison of differences in effector T cells and inflammatory factors between healthy controls (n = 20) and CHIKF patients (n = 34). **(A)** Mann–Whitney U test analysis results of effector cells such as CD3^+^, CD3^+^CD8^+^, CD4^+/^CD8^+^ Ratio and NKT in the blood of the two groups. **(B)** is the independent sample t-test analysis result of pro-inflammatory cytokines such as IL-1β, IL-6, and IL-8 in the blood of the two groups. **(C)** is the independent sample t-test analysis result of anti-inflammatory cytokines such as IL-17A and IL-4 in the blood of the two groups. **(D)** is the independent sample t-test analysis result of interferons such as IFN-α and IFN-γ in the blood of the two groups. Data are presented as box-and-dot plots, where whiskers indicate the minimum and maximum values, and the horizontal line represents the mean.

Peripheral blood samples from CHIKF patients and healthy controls were analyzed to assess immunological alterations. Compared to controls, CHIKF patients demonstrated significant reductions in key effector T cell populations: CD3^+^ T cells, CD3^+^CD8^+^ T cells, and natural killer T (NKT) cells were downregulated by 12.4% (P = 0.033), 27.7% (P = 0.004), and 52.2% (P < 0.001), respectively (**Figure 1A**). Conversely, the CD4^+^/CD8^+^ ratio was significantly increased by 60.8% (P = 0.035) (**Figure 1A**). The populations of CD4^+^ cells and natural killer (NK) cells (CD3^-^CD16^+^CD56^+^) were also analyzed, but no significant difference was observed between patients and healthy controls (**Supplementary Table 1**).

Concurrently, a marked pro-inflammatory cytokine response was observed. Levels of IL-1β, IL-6, and IL-8 were substantially elevated by 193.7% (P < 0.001), 256.2% (P = 0.002), and 950.9% (P < 0.001), respectively (**Figure 1B**). In contrast, the anti-inflammatory cytokines IL-4 and IL-17A were significantly downregulated by 39.0% (P = 0.005) and 32.9% (P < 0.001) (**Figure 1C**). The increased levels of IFN-α, IFN-γ in sera of the patients were observed, indicating antiviral responses were activated by CHIKV (**Figure 1D**). Among the cytokines we analyzed, the levels of IL-2, IL-10, TNF-α, IL-5, and IL-12 p70 in sera of patients were not significantly different from healthy controls (**Supplementary Table 1**).

Therefore, CHIKV infection establishes a dual immunopathogenic state characterized by reduction of effector T cells and a marked overexpression of pro-inflammatory cytokines, most notably IL-1β, IL-6, and IL-8.

### T cell dysregulation is associated with clinical symptoms in CHIKF patients

Clinical analysis of a cohort of 34 CHIKF patients revealed the following symptom profile: fever was present in 31 patients (duration 1–8 days), arthralgia in 27 patients (affecting ankles, knees, wrists, distal limb joints, fingers, elbows, and shoulders), and rash in 22 patients. Other reported symptoms included dizziness, headache, fatigue, myalgia, chest tightness, mild dyspnea, and chills.

**Figure 2:**
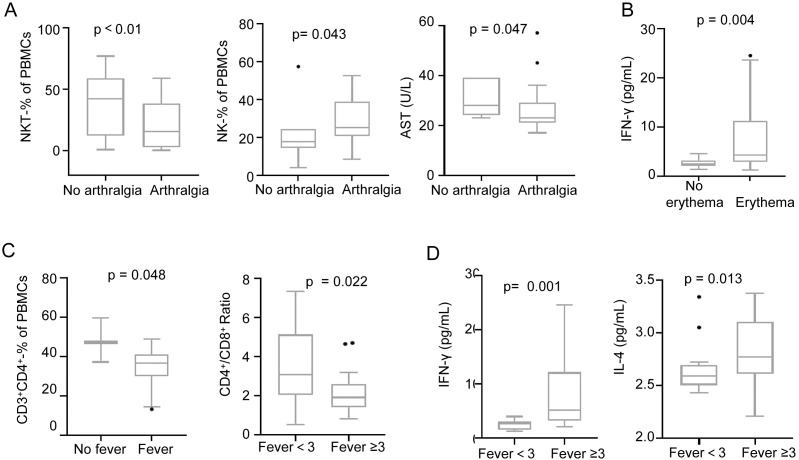
Association of clinical symptoms with immune cells and inflammatory factors in CHIKF patients. CHIKF patients (n=34) were stratified by the presence of key clinical symptoms (arthralgia, erythema, fever). Mann–Whitney U test were performed to compare effector T cells and cytokine levels between groups. **(A)** Analysis of NKT cells, IFN-γ, IL-2, and IL-5 levels between patients without arthralgia (n=7) and with arthralgia (n=27). **(B)** Analysis of IFN-γ and alanine aminotransferase (ALT) levels between patients without rash (n=12) and with rash (n=22). **(C)** Analysis of CD3^+^CD4^+^ T cells and the CD4^+^ ratio between patients without fever (n=3) and with fever (n=31), as well as between patients with fever duration <3 days (n=15) and ≥3 days (n=19). **(D)** Analysis of IFN-γ, IL-4, IL-5, and ALT levels between patients with fever duration <3 days and ≥3 days. Data are presented as box-and-dot plots (whiskers: min/max; line: mean).

Compared to patients without arthralgia, those with arthralgia exhibited significant reduction in NKT cells (-50.4%, p < 0.01), increment in NK cells (+36.6%, p = 0.043), along with reduction in serum AST levels (-14.3%, p = 0.047) (**Figure 2A**). No significant differences were observed in CD3^+^CD4^+^ T cells, IL-1β, IL-6, or IL-8 between groups.

Patients with rash showed markedly upregulated IFN-γ (+193.1%, P = 0.004) compared to those without rash (**Figure 2B**), while CD3^+^CD4^+^ T cells, IL-1β, IL-6, and IL-8 levels remained comparable.

Febrile patients had lower CD3^+^CD4^+^ T cell levels than afebrile patients (-27.8%, p = 0.048) (**Figure 2C**). Among febrile patients, those with fever lasting ≥3 days displayed a decreased CD4^+^/CD8^+^ ratio (-38.6%, p = 0.022) (**Figure 2C**), elevated serum IFN-γ (+247%, p < 0.01) and IL-4 (+7.9%, p = 0.013) compared to those with fever <3 days (**Figure 2D**). Pro-inflammatory cytokines (IL-1β, IL-6, IL-8) showed no significant variation with fever status.

These findings indicate that effector T cell dysregulation is closely associated with the appearance of arthralgia and fever in CHIKF patients, highlighting symptom-specific immunopathological signatures.

### Basic diseases and advanced age exacerbate immune dysregulation in CHIKF patients

**Figure 3:**
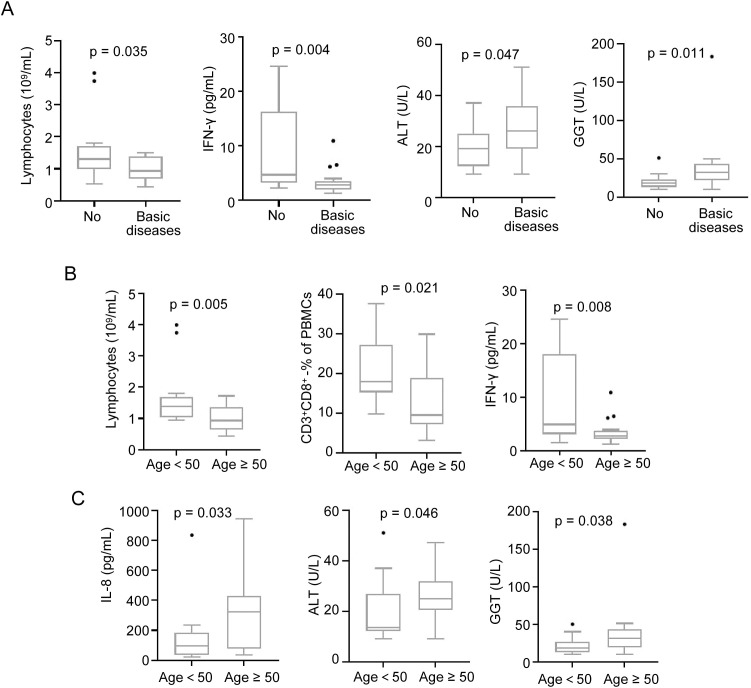
Impact of underlying diseases and age on immune cells and cytokines in CHIKF patients. CHIKF patients (n=34) were stratified by the presence of underlying comorbidities and by age (<50 vs. ≥50 years). Mann–Whitney U test were used for analysis. **(A)** Comparison of immune cell levels (lymphocytes, IFN-γ, ALT, GGT) between patients without (n=16) and with (n=18) underlying diseases. **(B)** Comparison of immune cell levels (lymphocytes, CD3^+^CD8^+^ T cells, IFN-γ) between patients aged <50 years (n=14) and ≥50 years (n=20). **(C)** Comparison of cytokine levels (IL-8, ALT, GGT) between the age groups. Data are presented as box-and-dot plots (whiskers: min/max; line: mean).

Analysis of the 34 CHIKF patients (15 females, 19 males; 14 aged <50 years, 20 aged ≥50 years) showed that 18 patients had underlying basic diseases, including diabetes, hypertension, hyperlipidemia, and hepatitis. Immunological comparison between CHIKF patients with and without basic diseases revealed that those with basic diseases exhibited significantly lower levels of lymphocytes (-35.7%, p = 0.035) (**Figure 3A**). Furthermore, IFN-γ expression was downregulated by 61.9% (p = 0.004), while serum ALT and glutamyltransferase (GGT) were elevated by 41.0% (p = 0.047) and 96.6% (p = 0.011), respectively (**Figure 3A**). In contrast, levels of pro-inflammatory cytokines IL-1β, IL-6, IL-8 and the other detected factors showed no significant differences between the two groups (**Table 1**).

**Table 1:** Univariate basic disease analysis of clinical testing indicators in patients with chikungunya fever.

Basic disease		Yes (n = 18)	No (n = 16)	Difference significance
Age (years)		61.90 ± 20.08	36.94 ± 20.39	**P = 0.001**
Gender (man/all)		11/18 (61.1%)	8/16 (50.0%)	P = 0.515
Duration of fever (days)		2.72 ± 2.16	3.60 ± 2.23	P = 0.261
Arthralgia (yes/all)		16/18 (99.9%)	11/16 (68.8%)	P = 0.147
Erythema (yes/all)		9/18 (50.0%)	13/16 (81.3%)	P = 0.057
Blood cell	Leukocyte (*10^9/mL)	4.4 (2.9, 5.1)	4.2 (3.3, 4.6)	P = 0.286
	Neutrophil (*10^9/mL)	2.6 (1.3, 3.3)	2.1 (1.5, 3.1)	P = 0.125
	Lymphocyte (*10^9/mL)	0.9 (0.7, 1.4)	1.2 (1.0, 1.7)	**P = 0.013**
	CD3^+^ cells (%)	52.3 (43.9, 69.7)	62.2 (53.5, 69.2)	**P = 0.033**
	CD3^+^ CD4^+^ cells (%)	36.6 (22.2, 40.3)	39.8 (34.9, 46.0)	P = 0.83
	CD3^+^ CD8^+^ cells (%)	9.8 (8.7, 25.1)	17.5 (14.6, 24.4)	**P = 0.004**
	CD4^+^/CD8^+^	2.4 (1.5, 4.7)	2.1(1.6, 3.2)	**P = 0.035**
	CD3^-^CD16^+^CD56^+^ cells (%)	25.0 (18.5, 38.0)	24.3(18.6, 35.6)	P = 0.69
	NKT cells (%)	14.2 (1.84, 23.8)	42.2 (12.5, 56.9)	**P < 0.001**
Cytokine	IL-2 (pg/mL)	1.0 (0.7, 1.0)	0.8 (0, 1.2)	P = 0.569
	IL-4 (pg/mL)	2.6 (2.5, 2.7)	2.8 (2.6, 3.1)	**P < 0.001**
	IL-6 (pg/mL)	4.9 (2.7, 15.2)	8.8 (4.6, 14)	**P = 0.002**
	IL-10 (pg/mL)	4.6 (3.4, 5.3)	4.3 (3.7, 5.8)	P = 0.485
	TNF-α (pg/mL)	1.5 (1.2, 2.1)	1.4 (1.1, 1.8)	P = 0.244
	IFN-γ (pg/mL)	2.7(1.8, 3.6)	4.6 (3.1, 12.1)	**P = 0.002**
	IL-17A (pg/mL)	0.4 (0.2, 0.6)	0.5 (0.4, 0.7)	**P = 0.005**
	IL-1β (pg/mL)	4.2 (2.2, 5.5)	3.3 (2.2, 4.2)	**P < 0.001**
	IL-5 (pg/mL)	1.4 (1.3, 1.5)	1.2 (0.1, 1.7)	P = 0.103
	IL-12p70 (pg/mL)	0.4 (0.2, 1.0)	0.7 (0.4, 1.0)	P = 0.754
	IFN-α (pg/mL)	1.5 (0.9, 2.2)	1.8 (0.8, 3.5)	**P = 0.002**
	IL-8 (pg/mL)	234 (81, 381)	104 (54, 424)	P = 0.372
Serum factor	ALT (U/L)	27 (18, 36)	20 (13, 25)	**P = 0.016**
	AST (U/L)	23 (22, 32)	24 (21, 29)	P = 0.458
	GGT (U/L)	32 (22, 43)	18 (13, 23)	**P = 0.033**
	CRP (mg/L)	23 (8, 34)	12 (3, 35)	P = 0.182

Data are presented as median ± standard error of the median (IQR). Gender, arthralgia, and erythema were analyzed with Chi-square test, and other indicators were analyzed with Mann–Whitney U test. Bold font for P-values <0.05.

Age-related analysis demonstrated that patients aged ≥ 50 years had reduced lymphocyte and CD3^+^CD8^+^ T cell levels compared to those under 50 years (-40.7% and -34.9%, respectively) (**Figure 3B**), along with decreased IFN-γ (-64.0%, p = 0.008) (**Figure 3B**). Concurrently, pro-inflammatory cytokine IL-8, serum ALT and GGT were elevated by 69.4% (p = 0.033), 29.7% (p = 0.046) and 73.5% (p = 0.038), respectively (**Figure 3C**), whereas IL-1β and IL-6 levels remained comparable between age groups (**Supplementary Table 2**).

These results indicate that pre-existing basic diseases and advanced age exacerbate immune dysregulation and liver injury in CHIKF patients.

### Male CHIKF patients exhibit heightened risk of immune dysregulation and inflammation

**Figure 4:**
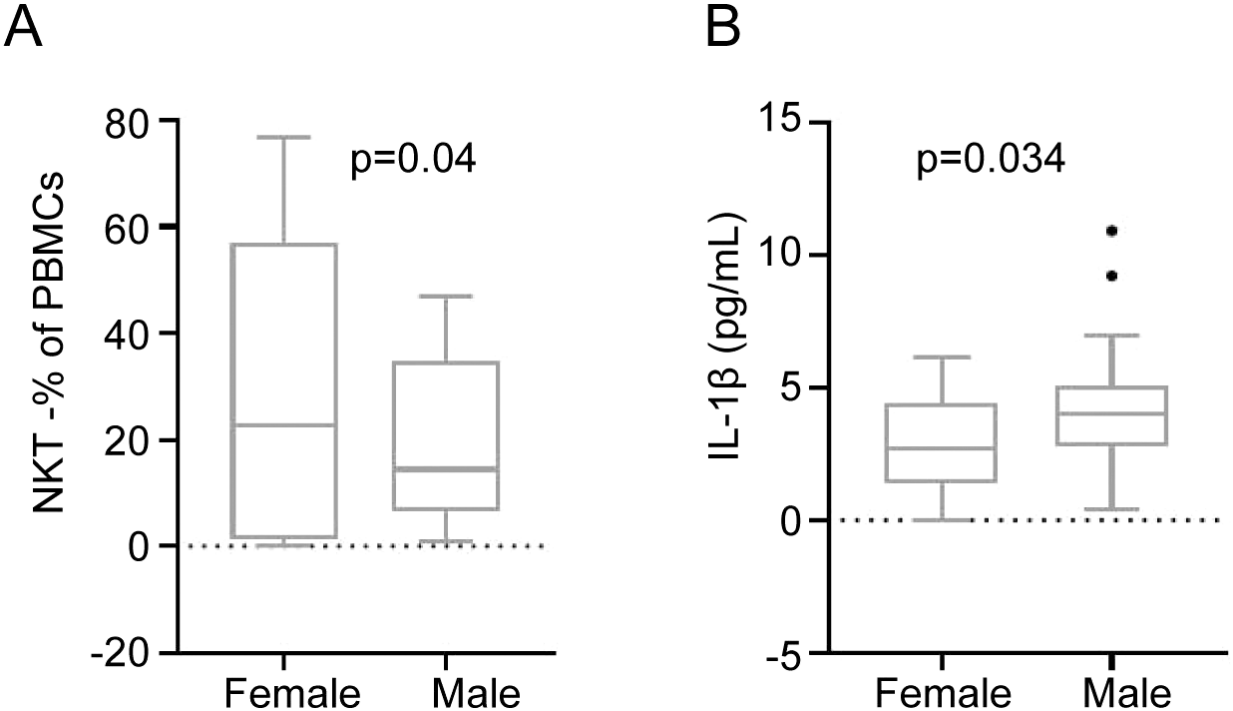
Analysis of differences in immune cells and pro-inflammatory factors between genders in CHIKF patients. The statistical analysis of NKT **(A)** and IL-1β **(B)** in 15 females and 19 males of the CHIKF patients. The data is presented in boxes and dots, with bars representing the maximum and minimum values, and the average value is highlighted as a line.

Analysis of the cohort comprising 19 male and 15 female CHIKF patients revealed significant sex-based immunological differences. Compared to female patients, males demonstrated a further reduction in NKT cell levels (-40.9%, P = 0.04) (**Figure 4A**) and a significant elevation of the pro-inflammatory cytokine IL-1β (+51.0%, P = 0.034) (**Figure 4B**). In contrast, no significant differences were observed between sexes in the expression levels of CD4^+^ T cells, CD8^+^ T cells, or the other inflammatory cytokines (**Table 2**).

**Table 2:** Univariate gender analysis of clinical testing indicators in patients with chikungunya fever.

Gender		Male (n = 19)	Female (n = 15)	Difference significance
Age (years)		46.32 ± 25.03	55.07 ± 21.50	P = 0.281
Basic disease (yes/all)		11/19 (57.8%)	7/15 (46.7%)	P = 0.730
Duration of fever (days)		3.33 ± 2.35	2.87 ± 2.07	P = 0.467
Arthralgia (yes/all)		16/19 (84.2%)	11/15 (73.3%)	P = 0.672
Erythema (yes/all)		13/19 (68.4%)	9/15 (60.0%)	P = 0.724
Blood cell	Leukocyte (*10^9/mL)	3.8 (2.9, 4.6)	3.8 (2.9, 4.6)	P = 0.098
	Neutrophil (*10^9/mL)	2.6 (2.1, 3.3)	1.6 (1.2, 3.1)	P = 0.137
	Lymphocyte (*10^9/mL)	1.1 (0.8, 1.4)	1.2 (0.9, 1.6)	P = 0.243
	CD3^+^ cells (%)	62.2 (49.9, 69.3)	59.7 (50.4, 67.7)	P = 0.418
	CD3^+^ CD4^+^ cells (%)	36.8 (29.9, 40.8)	36.6 (31.5, 45.7)	P = 0.122
	CD3^+^ CD8^+^ cells (%)	15.2 (9.2, 25.1)	17.0 (8.6, 24.4)	P = 0.383
	CD4^+^/CD8^+^	2.1 (1.5, 4.2)	2.5 (1.8, 4.6)	P = 0.401
	CD3^-^CD16^+^CD56^+^ cells (%)	24.2 (18.6, 38.0)	25.0 (18.6, 35.6)	P = 0.489
	NKT cells (%)	14.4 (6.8, 31.6)	22.6 (1.3, 56.9)	**P = 0.004**
Cytokine	IL-2 (pg/mL)	1.0 (0.3, 1.2)	0.9 (0, 1.1)	P = 0.317
	IL-4 (pg/mL)	2.7 (2.5, 3.0)	2.7 (2.6, 3.1)	P = 0.446
	IL-6 (pg/mL)	6.4 (3.0, 14.8)	7.9 (3.9, 14.8)	P = 0.397
	IL-10 (pg/mL)	4.6 (3.4, 5.6)	4.2 (3.8, 5.4)	P = 0.169
	TNF-α (pg/mL)	1.4 (1.1, 1.8)	1.4 (1.3, 2.1)	P = 0.338
	IFN-γ (pg/mL)	3.1 (2.1, 4.9)	3.2 (2.7, 10.9)	P = 0.294
	IL-17A (pg/mL)	0.4 (0.2, 0.7)	0.5 (0.4, 0.6)	P = 0.395
	IL-1β (pg/mL)	4.0 (2.8, 5.4)	2.7 (1.3, 4.4)	**P = 0.034**
	IL-5 (pg/mL)	1.3 (0.4, 1.5)	1.5 (0.2, 1.7)	P = 0.198
	IL-12 P70 (pg/mL)	0.5 (0.2, 0.9)	0.7 (0.4, 1.1)	P = 0.285
	IFN-α (pg/mL)	1.5 (0.7, 1.9)	2.1 (1.0, 3.2)	P = 0.290
	IL-8 (pg/mL)	219 (61, 373)	100 (55, 424)	P = 0.348
Serum factor	ALT (U/L)	25 (14, 35)	22 (14, 25)	P = 0.188
	AST (U/L)	23 (22, 30)	24 (21, 28)	P = 0.254
	GGT (U/L)	31 (21, 43)	19 (15, 25)	P = 0.477
	CRP (mg/L)	15.4 (6.2, 38.9)	26.7 (3.2, 32.8)	P = 0.226

Data are presented as median ± standard error of the median (IQR). Basic disease, arthralgia, and erythema were analyzed with Chi-square test, and other indicators were analyzed with Mann–Whitney U test. Bold font for P-values <0.05.

These findings indicate that male CHIKF patients experience more pronounced NKT cell depletion and greater upregulation of IL-1β, suggesting an elevated risk of immune dysregulation and inflammatory response compared to female patients.

### The induction of pro-inflammatory cytokines in patients with CHIKF was correlated with abnormal neutrophil and total leukocyte counts but not with dysregulated T cells

**Figure 5:**
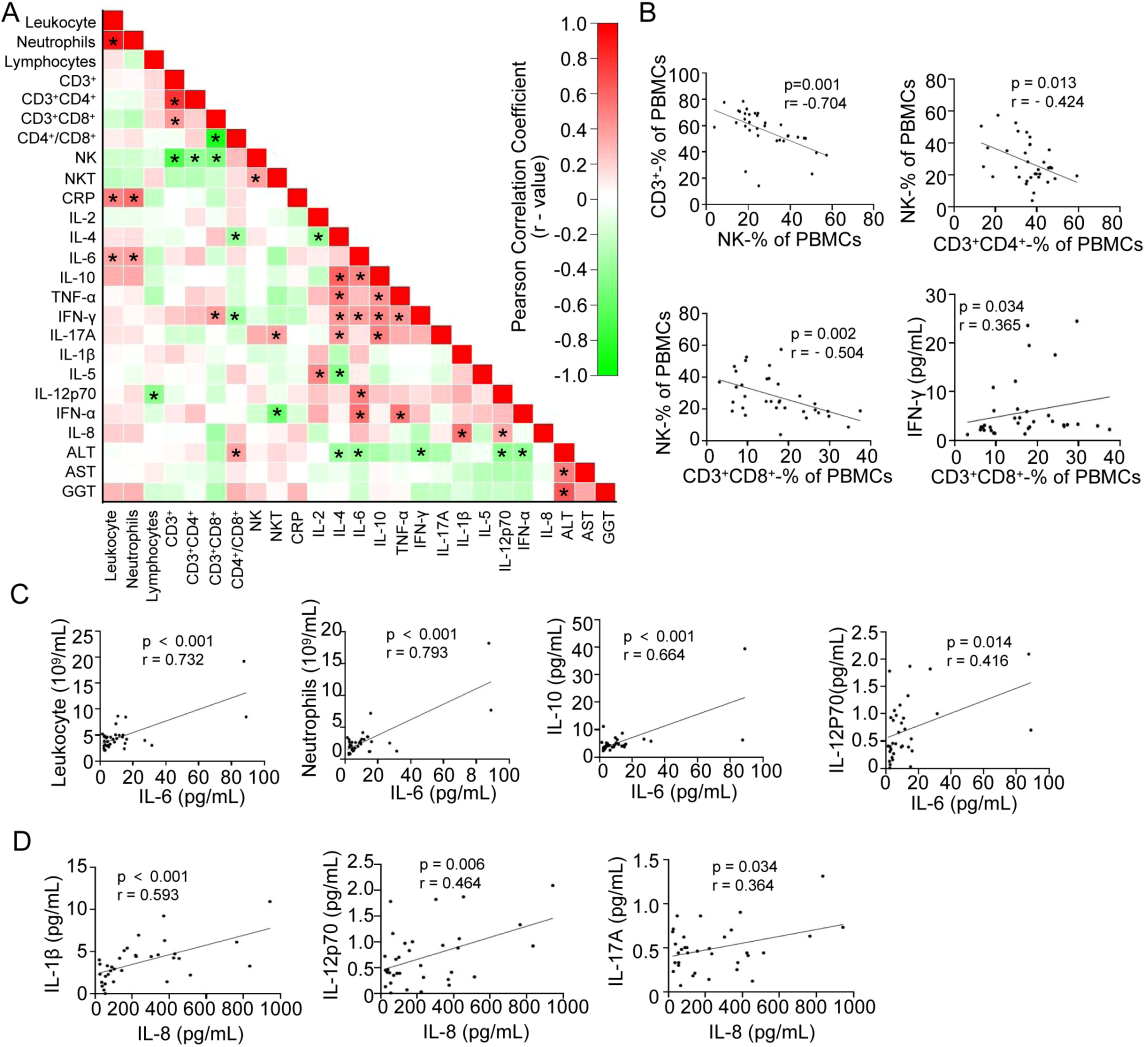
Spearman correlation analysis of immune cells and pro-inflammatory factors in CHIKF patients. **(A)** The heat map shows the Spearman correlation network for clinical liver function, immune cells, cytokines and other related parameters of CHIKF patients, including all BAL samples of n = 34 CHIKF patients tested (above heat map). Red indicates a positive correlation (R > 0); green indicates a negative correlation (R < 0). Cells are highlighted for associations with *p < 0.05. **(B-G)** Individual Spearman correlation plots highlighting specific relationships: **(B)** between effector NK cells and CD3^+^ T cells, CD3^+^CD4^+^ T cells; and between CD3^+^CD8^+^ T cells and NK cell levels, IFN-γ. **(C)** between IL-6 and leukocyte, neutrophil, IL-10 and IL-12p70 levels. **(D)** between IL-8 and IL-1β, IL-12p70, and IL-17A levels. r, correlation coefficient; p, p-value from Spearman correlation analysis.

To investigate associations between immune and inflammatory markers in CHIKF, we performed Spearman correlation analysis on the tested indicators. No significant correlation was found between dysregulated T cell subsets (CD4^+^, CD8^+^, or NKT cells) and pro-inflammatory cytokine levels (IL-1β, IL-6, or IL-8) (**Figure 5A**).

Analysis further revealed that NK cells were negatively correlated with between CD3^+^, CD4^+^ and CD8^+^ T cells, and that CD8^+^ T cell subsets were moderately positively correlated with serum IFN-γ (**Figure 5B**). Pro-inflammatory cytokine IL-6 showed positive correlations with neutrophil counts, and total leukocyte counts (**Figure 5C**). Additionally, IL-6 levels were positively correlated with IL-10 and IL-12p70 (**Figure 5C**), while IL-8 levels showed positive correlations with IL-1β, IL-12p70, and IL-17A (**Figure 5D**). These correlation patterns likely reflect functional interactions within immune and inflammatory networks during CHIKV infection.

## Discussion

In this study, peripheral blood samples were analyzed by flow cytometry and multiplex cytokine assays. Compared with the healthy control group, patients with chikungunya fever (CHIKF) exhibited significant reductions in the frequencies of CD3^+^ T cells, CD8^+^ T cells, and NKT cells. These findings suggest that CHIKV may dysregulate the immune function of infected individuals by targeting T cell populations. Previous studies have indicated a strong association between CD4^+^ T cells and arthralgia (Teo et al., 2017; Agarwal et al., 2025b). We observed that the proportion of NKT cells in peripheral blood mononuclear cells (PBMCs) was significantly lower in patients with arthralgia than in those without (**Figure 2A**), whereas no significant differences were found in CD4^+^ or CD8^+^ T cell frequencies (data not shown). Furthermore, a decrease in CD4^+^ T cell frequency was associated with the presence of fever (**Figure 2C**); however, no differences in CD4^+^, CD8^+^, or NKT cells were detected between patients with and without rash (data not shown). In addition, plasma levels of IFN-γ were significantly related with rash and prolonged fever (**Figures 2B, D**). Given that IFN-γ is primarily produced by effector T cells *in vivo (*Hwang et al., 2025), this result further supports the important role of T cells in the pathogenesis of CHIKV infection. To elucidate how CHIKV infection drives disease through T-cell-mediated mechanisms, more detailed investigations into T-cell subsets - such as Th1, Th2, and Th17 among CD4^+^ T cells, and Tc1, Tc2, and Tc9 among CD8^+^ T cells - are warranted. One limitation of this part of the study is the small sample size in certain comparison groups, such as the number of data points in the groups without fever (n = 3) and without joint pain (n = 7). Although statistical conclusions were drawn, the findings may lack sufficient power and should be interpreted with caution. As such, these results are presented as exploratory observations for reference only.

Our findings suggest that both pre-existing diseases and advanced age correlate with a more pronounced degree of immune dysregulation (**Figures 3A, B**). However, the conclusion could not be drawn from univariate analysis due to the significant age difference observed between patients with underlying diseases and those without. This constitutes a limitation of the present study, and a larger patient cohort would be required to perform separate univariate analyses for age and pre-existing conditions. Studies on SARS-CoV-2 infection have shown that elderly individuals and those with underlying medical conditions face a higher risk of severe illness, which is accompanied by more pronounced suppression of type I interferon responses (Hurst et al., 2025; Petrov et al., 2025). However, in our study, more pronounced suppression of type I interferon was not observed in older individuals or those with underlying medical conditions infected with Chikungunya virus. Instead, we observed suppression of IFN-γ which is consistent with the observed inhibition of CD8+ T cells (**Figure 3B**).

Hepatic dysfunction is a recognized clinical manifestation of chikungunya virus infection (Costa et al., 2024; Wang and Wang, 2025). To assess liver injury in infected individuals, we measured plasma levels of three hepatocellular enzymes - aspartate aminotransferase (AST), alanine aminotransferase (ALT), and gamma-glutamyl transferase (GGT). Among the 34 patients included, 16 without pre-existing liver disease exhibited signs of hepatocellular damage following CHIKV infection. Correlation analysis demonstrated a significant association between serum ALT and both IFN-α and IL-12p70 (**Supplementary Figure 2**). Considering the established roles of IFN-α and IL-12p70 in antiviral immunity (Delale et al., 2005; Kelvin et al., 2011; Bezerra et al., 2023), we propose that hepatic impairment in these patients may be related to the host antiviral immune response. Furthermore, we observed that absence of arthralgia was associated with higher AST levels (**Figure 2A**). These findings provide further evidence supporting the link between liver injury and antiviral immunity during CHIKV infection. Similar to Chikungunya virus, cytomegalovirus (CMV) and Epstein-Barr virus (EBV) infections can also cause liver injury (Du et al., 2026). Investigating the differences and underlying mechanisms of liver injury induced by Chikungunya virus compared to other viruses represents a meaningful and valuable direction for future research.

This study indicated that male patients are at a higher risk of immune response dysregulation and inflammatory imbalance compared to female patients. Specifically, male patients exhibited a further reduction in NKT cell levels (**Figure 4A**) and a significant elevation in the pro−inflammatory cytokine IL−1β (**Figure 4B**). In contrast, no significant differences were observed between genders in the frequencies of CD4^+^ T cells or CD8^+^ T cells, or in the levels of other inflammatory cytokines (**Table 2**). These findings suggest that male patients with CHIKF experience more pronounced NKT cell depletion and upregulation of IL−1β than their female counterparts. Studies in patients with dengue fever and COVID-19 also showed that men were more susceptible, and in both cases, the activity of T cells was inhibited in the patients (Löhr et al., 2023; Musa et al., 2025). The observed gender differences in the pathogenicity of CHIKV infection warrant further investigation. Future studies could explore the influence of Y−chromosome−associated genes, sex hormones, and other gender−related factors in disease progression.

The correlation analysis of the detected indicators may help elucidate their interactions within the immune and inflammatory networks. IL-1β, IL-6, and IL-8 showed positive correlations with neutrophil counts, white blood cell counts, and C−reactive protein (CRP) levels, consistent with their established roles in antiviral innate immunity and inflammation (Chen et al., 2013; Deckx et al., 2016; Bezerra et al., 2023). Furthermore, CD3^+^ T cells were found to be negatively correlated with NK cells (**Figure 5B**), a pattern that may reflect the transition from early innate immune responses to subsequent adaptive immunity during infection. This also suggests that virus-induced dysregulation of T cells could potentially influence innate immune and inflammatory pathways. Notably, no significant correlation was observed between the altered T-cell subsets (CD4^+^, CD8^+^, or NKT cells) and levels of the pro-inflammatory cytokines IL-1β, IL-6, or IL-8 (**Figure 5A**), implying that these cytokines may predominantly originate from innate immune sources.

Based on the analysis of 34 chikungunya fever cases and 20 healthy blood samples, we draw the following conclusions: 1) Dysregulation of T cells and cytokines is closely associated with the pathogenicity of CHIKV infection; 2) Male patients exhibit a higher risk of T−cell subset reduction and inflammatory imbalance than females; 3) Activation of the antiviral immune response during CHIKV infection is accompanied by liver injury. Furthermore, this study highlights several directions for future research, including more detailed T−cell subset analysis, investigation of gender−based differences, liver−related antiviral mechanisms, and the activation pathways of specific cytokines. It should be noted that this study has certain limitations - all analyses were conducted using univariate methods, which resulted in insufficient control for confounding variables.

## Data Availability

The original contributions presented in the study are included in the article/**Supplementary Material**. Further inquiries can be directed to the corresponding authors.
