# Supplementary material for: Dysregulated T cell responses and inflammatory cytokine profiles in patients with acute chikungunya fever: a study from Guangdong, 2025
Source: Front Cell Infect Microbiol. 2026 Apr 7;16:1774254. doi: 10.3389/fcimb.2026.1774254 (PMC13095717; doi:10.3389/fcimb.2026.1774254)
Supplement: Supplementary file 1 [file Table1.docx]

**Supporting Information**

**Dysregulated T Cell Responses and Inflammatory Cytokine Profiles in Patients with Acute Chikungunya Fever: A Study from Guangdong, 2025**

**Running Title:** CHIKV-induced Immune Dysregulation

Mingya Xiao^1, #^, Zijing Liu^2, 3, #^,Si Wang^2, #^, Qian Yi^2^, E-ying Lu^1^, Ruirong Xu^1^,Wei Li^2, *^, Yongkui Li^2, 3, *^, Wenli Li^1, *^

^1^Department of Infectious Diseases, The Affiliated Guangdong Second Provincial General Hospital of Jinan University, Guangzhou, China.

^2^Department of Immunology and Microbiology, Institute of Medical Microbiology, College of Life Science and Technology, Jinan University, Guangzhou, China

^3^Key Laboratory of Viral Pathogenesis & Infection Prevention and Control (Jinan University), Ministry of Education, Guangzhou, China

^#^These authors contributed equally to this work.

^*^Correspondence: Wenli Li, Email: liwenli-1978@163.com; or Yongkui Li, lyk070@jnu.edu.cn; or Wei Li, Email: 515146023@qq.com

**Supplementary Figure 1**

**
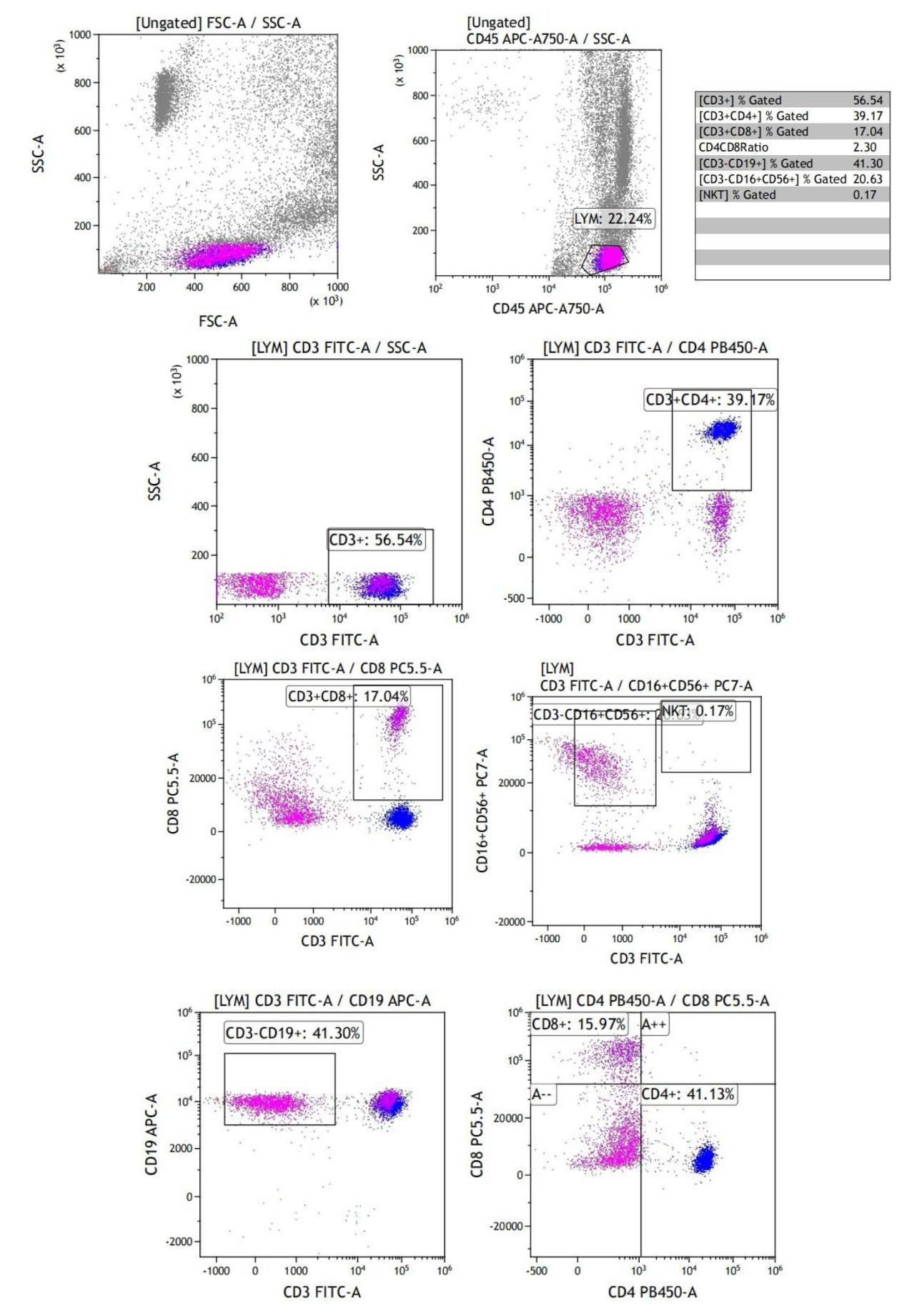
**

**Supplementary Figure 2**


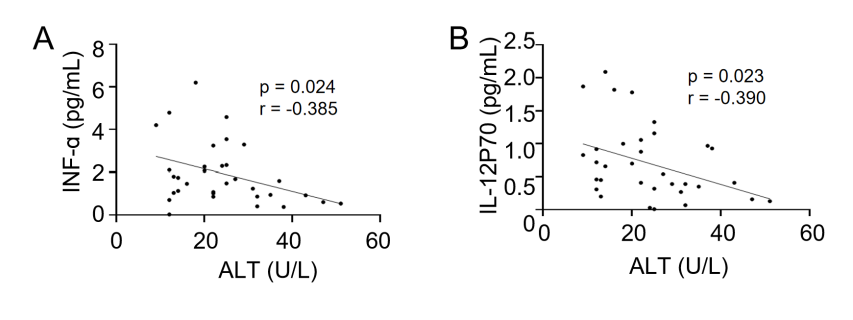


**Supplementary Figure Legend**

**Supplementary Figure 1** The flow cytometry analysis of 1 patient sample was shown, that displayed the gating strategy, the quality control and states after compensation.

**Supplementary Figure 2** (A) Pearson correlation analysis between ALT and INF-α levels; (B) Pearson correlation analysis between ALT and IL12p70 levels.

**Supplementary Table 1.** Analysis of clinical detection indicators of patients with Chikungunya fever and healthy controls.

| Sample category | | Healthy  (n = 20) | Chikungunya fever infection  (n = 34) | Difference significance |
| --- | --- | --- | --- | --- |
| Gender (man/all) | | 12 / 20 (60%) | 19 / 34 (55.9%) | p = 0.768 |
| Basic disease (yes/all) | | 12 / 20 (60%) | 18 / 34 (52.9%) | p = 0.614 |
| Age (years) | | 45.30 ± 17.45 | 50.18 ± 23.60 | p = 0.426 |
| Blood cells | CD3^+^ cells (%) | 62.8 (60.9, 70.3) | 59.3 (50.1, 68.7) | p **= 0.033** |
|  | CD3^+^ CD4^+^ cells (%) | 37.7 (34.8, 42.6) | 36.7 (30.7, 42.0) | p = 0.830 |
|  | CD3^+^ CD8^+^ cells (%) | 23.0 (18.2, 28.8) | 16.4 (9.2, 23.9) | p **= 0.004** |
|  | CD4^+^/CD8^+^ ratio | 1.7 (1.5, 2.3) | 2.3 (1.5, 4.0) | p **= 0.035** |
|  | CD3^-^CD16^+^CD56^+^ cells (%) | 25.5 (17.0, 32.9) | 24.5 (18.6, 37.3) | p = 0.694 |
|  | NKT cells (%) | 56.2 (41.6, 62.2) | 17.6 (5.5, 43.1) | p **＜ 0.001** |
| Cytokines | IL-2 (pg/mL) | 0.5 (0, 1.2) | 0.9 (0, 1.1) | p = 0.569 |
|  | IL-4 (pg/mL) | 3.5 (3.1, 5.6) | 2.7 (2.5, 3.1) | p **＜ 0.001** |
|  | IL-6 (pg/mL) | 3.4 (0.8, 6.2) | 7.2 (3.2, 14.2) | p **= 0.002** |
|  | IL-10 (pg/mL) | 4.7 (3.4, 9.4) | 4.6 (3.7, 5.9) | p = 0.485 |
|  | TNF-α (pg/mL) | 1.4 (1.2, 1.6) | 1.5 (1.2, 1.9) | p = 0.244 |
|  | IFN-γ (pg/mL) | 1.9 (1.3, 2.9) | 3.2 (2.4, 6.0) | p **= 0.002** |
|  | IL-17A (pg/mL) | 0.7 (0.5, 0.9) | 0.5 (0.3, 0.6) | p **= 0.005** |
|  | IL-1β (pg/mL) | 0.7 (0, 2.3) | 3.4 (2.2, 4.6) | p **＜ 0.001** |
|  | IL-5 (pg/mL) | 0.7 (0, 1.5) | 1.4 (0.3, 1.6) | p = 0.103 |
|  | IL-12p70 (pg/mL) | 0.6 (0.4, 0.9) | 0.5 (0.3, 1.0) | p = 0.754 |
|  | IFN-α (pg/mL) | 0.8 (0.5, 1.0) | 1.5 (0.9, 2.6) | p **= 0.002** |
|  | IL-8 (pg/mL) | 17.0 (13.6, 37.2) | 150 (55.9, 378.6) | p **＜ 0.001** |

Note：Data are presented as median ± standard error of the median (IQR). Gender and basic disease were analyzed with Chi-square test, and other indicators were analyzed with Mann–Whitney U test.

**Supplementary Table 2.** Univariate age analysis of clinical testing indicators in patients with chikungunya fever.

| Age | | < 50 years  (n = 14) | ≥ 50 years  (n = 20) | Difference significance |
| --- | --- | --- | --- | --- |
| Gender (man/all) | | 10 / 14 (71.4%) | 9 / 20 (45.0%) | P ＜ 0.001 |
| Basic disease (yes/all) | | 3 / 14 (21.4%) | 15 / 20 (75.0%) | ***P = 0.002*** |
| Duration of fever (days) | | 3.62 ± 1.94 | 2.80 ± 2.35 | P = 0.307 |
| Arthralgia (yes/all) | | 11 / 14 (78.6%) | 16 / 20 (80.0%) | P = 0.919 |
| Erythra (yes/all) | | 12 / 14 (85.7%) | 10 / 20 (50.0%) | **P = 0.032** |
| Blood cell | Leukocyte (*10^9/mL) | 4.2 (3.2, 4.8) | 4.2 (3.0, 5.1) | P = 0.713 |
|  | Neutrophil (*10^9/mL) | 2.0 (1.3, 3.1) | 2.6 (1.3, 3.4) | P = 0.522 |
|  | Lymphocyte (*10^9/mL) | 1.4 (1.0, 1.6) | 0.9 (0.6, 1.4) | **P = 0.005** |
|  | CD3^+^ cells (%) | 62.2 (51.2, 69.3) | 57.9 (49.1, 68.6) | P = 0.500 |
|  | CD3^+^ CD4^+^ cells (%) | 36.6 (30.4, 43.3) | 36.8 (31.5, 43.4) | P = 0.849 |
|  | CD3^+^ CD8^+^ cells (%) | 17.7 (15.1, 28.0) | 9.5 (7.2, 19.1) | **P = 0.021** |
|  | CD4^+^/CD8^+^ | 1.9 (1.4, 2.6) | 3.0 (1.8, 5.0) | P = 0.056 |
|  | CD3^-^CD16^+^CD56^+^ cells (%) | 24.7 (18.6, 37.2) | 24.2 (18.6, 36.9) | P = 0.624 |
|  | NK T cells (%) | 25.0 (9.5, 52.1) | 17.3 (2.8, 39.7) | P = 0.441 |
| Cytokine | IL-2 (pg/mL) | 1.0 (0, 1.2) | 0.9 (0, 1.1) | P = 0.620 |
|  | IL-4 (pg/mL) | 2.7 (2.6, 3.0) | 2.7 (2.5, 3.1) | P = 0.576 |
|  | IL-6 (pg/mL) | 6.4 (3.2, 12.9) | 8.8 (3.2, 5.5) | P = 0.780 |
|  | IL-10 (pg/mL) | 3.8 (3.2, 5.5) | 4.6 (4.0, 5.4) | P = 0.234 |
|  | TNF-α (pg/mL) | 1.4 (1.1, 1.7) | 1.6 (1.2, 2.1) | P = 0.234 |
|  | IFN-γ (pg/mL) | 4.6 (3.1, 14.9) | 2.8 (2.1, 3.8) | **P = 0.008** |
|  | IL-17A (pg/mL) | 0.5 (0.3, 0.7) | 0.4 (0.2, 0.6) | P = 0.506 |
|  | IL-1β (pg/mL) | 3.3 (2.2, 4.3) | 4.2 (2.2, 4.7) | P = 0.753 |
|  | IL-5 (pg/mL) | 1.3 (0.2, 1.6) | 1.5 (0.4, 1.6) | P = 0.944 |
|  | IL-12 p70 (pg/mL) | 0.5 (0.2, 0.8) | 0.7 (0.3, 1.3) | P = 0.162 |
|  | IFN-α (pg/mL) | 1.5 (0.6, 2.2) | 1.7 (0.3, 1.3) | P = 0.889 |
|  | IL-8 (pg/mL) | 84 (33, 198) | 340 (90, 431) | **P = 0.033** |
| Serum  factor | ALT ( U/L ) | 14 (12, 28) | 25 (20, 32) | **P = 0.046** |
|  | AST ( U/L ) | 23 (20, 30) | 25 (21, 28) | P = 0.731 |
|  | GGT ( U/L ) | 18 (13, 26) | 31 (19, 43) | **P = 0.038** |
|  | CRP ( mg/L ) | 12 (6, 33) | 23 (3, 35) | P = 0.636 |

Note：Data are presented as median ± standard error of the median (IQR). Gender, basic disease, arthralgia, and erythra were analyzed with Chi-square test, and other indicators were analyzed with Mann–Whitney U test.
